# Disseminated cryptococcosis and hepatitis C virus infection: A fatal co-infection

**DOI:** 10.18502/cmm.5.4.2163

**Published:** 2019

**Authors:** Ranjana Rohilla, Suneeta Meena, Neelam Kaistha, Anusha Krishna Raj, Pratima Gupta

**Affiliations:** Department of Microbiology, All India Institute of Medical Sciences, Rishikesh, Uttarakhand, India

**Keywords:** Cryptococcosis, Hepatitis C virus, Matrix-assisted laser desorption ionization- time of flight mass spectrometry

## Abstract

**Background and Purpose:**

We report a case of disseminated cryptococcosis in a treatment-naïve patient, incidentally diagnosed with hepatitis C virus (HCV) infection and renal parenchymal disease. The patient succumbed to death given the very late diagnosis of the disease.

**Case report:**

A 54-year-old male presented with the chief complaints of abdominal pain, chest pain, and phlegmy cough for a month. There was a past history of decreased urine output, lower limb swelling, and fever lasting for 15-20 days. After a general physical examination, the differential diagnosis of hepatitis C-related liver disease with hepatic encephalopathy, disseminated tuberculosis, and septic shock was made. Radiological examination revealed renal parenchymal disease on ultrasound abdomen and opacity with reticulonodular opacity in the bilateral lung zones. In laboratory investigations, serum reactive sample was tested for anti-HCV antibodies. In addition, *Cryptococcus var grubii* was identified in blood culture using the matrix-assisted laser desorption ionization-time of flight mass spectrometry (Bruker Daltonics, Germany). The patient succumbed to death before the initiation of any specific antifungal therapy.

**Conclusion:**

Cryptococcosis-HCV co-infection is a fatal condition with a fulminant course that might be difficult to treat.

## Introduction

*Cryptococcus*, a pathogenic capsulated fungal pathogen, causes a range of systemic infections. A normally functioning host immune system is capable of eliminating this infection or can sequester *Cryptococcus* into sites where it is kept under check by the host defense mechanisms. Clinically, this infection can range from asymptomatic colonization of the respiratory tract to widespread dissemination depending on the host immune factors. 

Cryptococcosis, which was once used to be a acquired immune deficiency syndrome defining illness, is now distributed globally and in a wide variety of hosts, ranging from those who are severely immunosuppressed to those who have an apparently healthy immune system [1]. *Cryptococcus* *neoformans*, encompassing var. *grubii* (serotype A) and var. *neoformans* (serotype D), and *C. gattii* (serotypes B and C) are two species of *Cryptococcus* that commonly induce disease in humans [2]. Isolation of *Cryptococcus* species from blood cultures (i.e., disseminated cryptococcosis) is a rare event, especially in patients with hepatic or renal pathologies. 

There is evidence regarding the higher prevalence of several infectious diseases, including cryptococcosis, among HCV-infected patients. This remains a significant complication in some cohorts of the patients, such as organ transplant recipients, drug users, patients with HCV/hepatitis B virus (HBV) infection, and those with renal diseases. It is important to highlight that the hematogenous dissemination of cryptococcosis to the brain is accompanied by a high mortality rate. Therefore, physicians must be aware of the possible isolation of *Cryptococcus* from blood in patients living with HCV/HBV infection [3]. Herein, we present a case of cryptococcosis who had an underlying HCV infection and renal parenchymal disease.

## Case report

A 54-year-old male, labourer by occupation, presented to the outpatient department of a tertiary care health institute with the chief complaints of abdominal pain, chest pain, and phlegmy cough for a month. He also complained of decreased urinary output and lower limb swelling for 3 days. In addition, he recalled a significant history of fever lasting for 15-20 days 4 months back and intake of antitubercular therapy. He was a known smoker; however, he did not drink alcohol. At the time of admission, he was disoriented to time, place, and person. 

**Figure 1:**
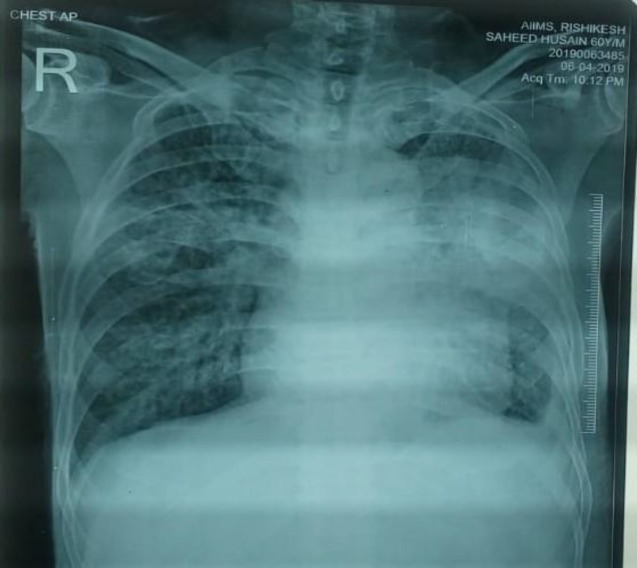
Chest X-ray showing heterogeneous opacity in the left upper lung zone with reticulonodular opacity in bilateral lung

Physical examination revealed the pulse of 113 bpm, blood pressure of 80/50 mmHg, respiratory rate of 20 per minute, and room air SpO_2_ of 98%. He was also detected with pallor, icterus, and bilateral pedal edema. In addition, the central nervous system examination showed flapping tremors. In the cardiovascular system examination, no added sound was heard. However, bilateral basal crept and occasional rhonchi were heard on chest auscultation. On the abdomen examination, abdominal distension was observed; furthermore, the liver was palpable and non-tender.

The chest X-ray demonstrated heterogeneous opacity in the left upper lung zone with reticulonodular opacity in the bilateral lung zones (Figure 1). Ultrasound abdomen was suggestive of grade II fatty liver changes with increased echogenicity; however, no focal lesion was seen. The kidneys were normal in size, the cortical echotexture appeared to be raised, and corticomedullary differentiation was maintained. Accordingly, the ultrasound was suggestive of bilateral grade I renal parenchymal disease with mild ascites.

Based on the results of the rapid lateral flow dipstick test, the patient had a reactive anti-HCV antibody test (Tulip Diagnostics, Hyderabad, Telangana). A complete hemogram revealed a total leukocyte count of 16,850 cells per mm^3^. On differential leukocyte count, neutrophils, lymphocytes, monocytes, and basophils were obtained as 77.2%, 19.2%, 1.6%, and 2.0%, respectively. The liver function tests revealed increased total bilirubin, direct bilirubin, indirect bilirubin, serum glutamic pyruvic transaminase, serum glutamic oxaloacetic transaminase, and alkaline phosphatase levels of 16.7 mg/dl, 11.59 mg/dl, 5.11 mg/dl, 47.5 IU/ml, 49.2 IU/ml, and 268.3 U/L, respectively. Furthermore, the prothrombin time, international normalized ratio, serum urea, serum creatinine, and uric acid were estimated at 17 sec, 1.37, 207.3 mg/dl, 4.33 mg/dl, and 9.3 mg/dl, respectively. Additionally, phosphate, calcium, potassium sodium, and chloride levels were measured at 8.0 mg/dl, 8.0 mg/dl, 5.1 mEq/l, 135.2 mEq/l, and 99 mEq/l, respectively.

**Figure 2A:**
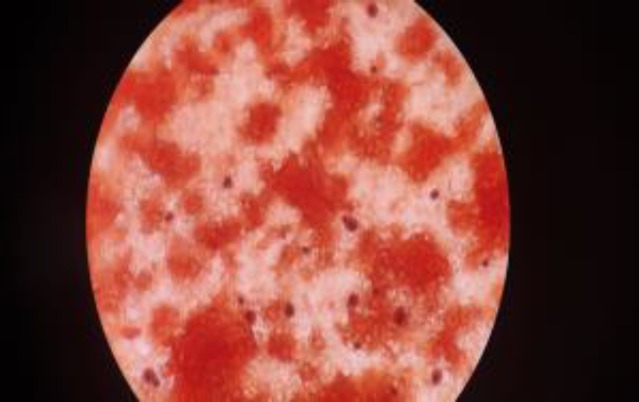
Capsulated gram positive budding yeast cells in blood culture

During the course of hospital stay, the patient’s blood pressure was constantly falling, and he was put on non-adrenaline and terlipressin for the maintenance of blood pressure. Differential diagnosis of HCV-related liver disease with hepatic encephalopathy, disseminated tuberculosis, and septic shock was made. He was started on ceftriaxone, azithromycin, vitamin K, and rifaximin. However, his condition did not improve even with a combination of antibiotics. Therefore, further escalation of antibiotics was planned.

Blood culture was sent to the microbiology lab for aerobic culture and sensitivity. The culture bottle signaled positive after 24 h of aerobic incubation in the BacT/ALERT 3D automated blood culture system (BioMerieux, Durham, N.C.). Variable sized (5-10 µm) encapsulated narrow-based budding yeast-like cells were seen in direct smear (Figure 2A). India ink preparation of blood culture sample aspirated from culture bottle showed budding yeast cells with a clear halo (Figure 2B). 

Creamy mucoid colonies were obtained in blood agar and Sabouraud dextrose agar after 48 h of aerobic incubation (Figure 2C). The isolate was identified by the MALDI biotyper (Bruker Daltonics, Germany) as *Cryptococcus var grubii* at a confidence interval of 2. The serum sample of the patient was also positive for lateral flow immunochromatographic test for cryptococcal antigen (CryptoPS, Biosynex, France) (Figure 2D). Patient’s attendants denied for antifungal treatment because of financial constraints, and they were willing to leave against medical advice. The patient expired the same day before the initiation of any antifungal therapy.

**Figure 2B:**
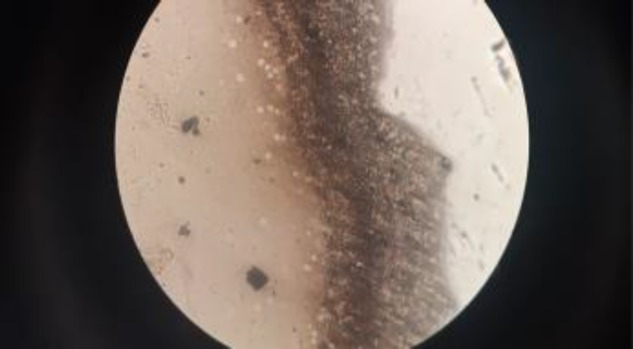
India ink preparation showing capsulated budding yeast cells

**Figure 2C:**
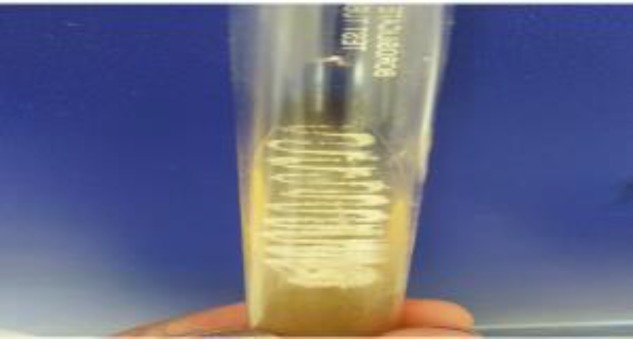
Creamy, mucoid colonies on Sabouraud dextrose agar after 48 hours of aerobic incubation

## Discussion

The patient present in the current study was a case of disseminated cryptococcosis with hepatitis C infection and renal parenchymal disease. Dissemination is due to serious defects in cell-mediated immune surveillance. *Cryptococcus *in blood indicates a poorer prognosis. When this species disseminates hematogenously, the central nervous system is the second commonly involved organ. Diagnosis of disseminated cryptococcosis is established based on positive cultures from any two organ sites (e.g., skin, central nervous system, peritoneum, and synovial fluid) or positive blood cultures [4, 5]. In our patient, it was not possible to perform lumbar puncture to confirm central nervous system involvement because of patient refusal to undergo any invasive procedure.

Literature shows that cryptococcosis remains a significant complication in patients who have a predisposing factor or underlying diseases, such as end-stage liver disease, renal insufficiency, sarcoidosis, and other conditions [1]. However, it can also affect a certain group of patients who are otherwise phenotypically normal, such as organ transplant recipients, drug users, and patients with HBV/HCV liver disease or renal disease [4]. Disseminated cryptococcal infection in patients suffering from hepatitis is rarely reported. El-Serag *et al.* concluded that patients suffering from HCV infection had a significantly higher prevalence of cryptococcal infections, compared to controls (0.4% vs 0.1%) [6,7]. Our case also highlights the importance of screening for the agents of viral hepatitis. The screening component of the National Viral Hepatitis Control Program can help to prevent the occurrence of such fatal infections in patients with HBV or HCV liver disease [8].

**Figure 2D:**
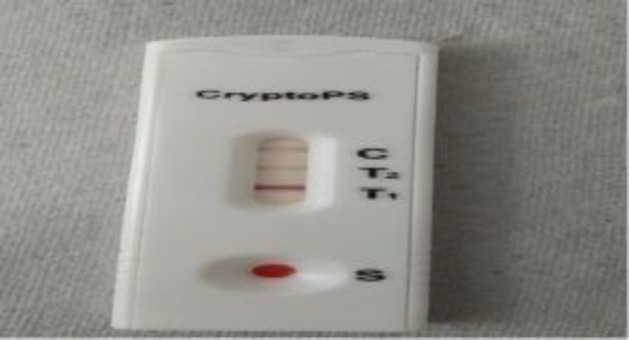
Positive lateral flow immunochromatographic test for cryptococcal, using serum sample

At the same time, there is a high incidence of invasive fungal infections by opportunistic pathogens, such as *Candida, Aspergillus, Mucor, Cryptococcus,* and *Histoplasma,* in patients with renal disease. Our patient had grade I renal parenchymal disease and also increased serum uric acid and serum creatinine. This might be another major risk factor in the above case for acquiring disseminated cryptococcosis [8, 9]. 

However, there were some limitations regarding the diagnosis and management procedures for our case. To comment upon the hepatitis C reactive status of this patient, owing to financial constraints, no molecular tests could be performed to detect viral load status, which could have helped in modifying the treatment regime. In addition, there was a history of antitubercular therapy intake; however, no specific investigation could be sought for tuberculosis (TB) because of the critically destabilizing vitals. The TB, if diagnosed, could be a major factor leading to mortality, apart from HCV infection and renal damage. Furthermore, an earlier diagnosis of disseminated cryptococcosis could have changed the disease course in our case. Aye *et al*. suggested that in patients with cryptococcal infections, the elongation of the diagnostic procedure may be the reason for higher mortality within 90 days [10, 11].

## Conclusion

Cryptococcosis-HCV co-infection is a fatal condition with a fulminant course that may be difficult to treat even when diagnosed early in the course of the disease. Such co-infections should be kept in mind in patients with sepsis-like presentations, who do not respond to broad standard antibiotic therapy. The National Viral Hepatitis Control Program may help in preventing such mortalities by facilitating the early diagnosis and management of viral hepatitis at all levels of healthcare.

## Ethical considerations

The written informed consent was obtained from the patients’ legal guardians regarding the publication of the patients’ images and other clinical information in the journal. Moreover, the legal guardians were informed of the confidentiality of data; however, the anonymity could not be guaranteed.

## Author’s contribution

R.R. performed the literature search, data analysis, and first draft of manuscript and figures. –S.M. carried out the subsequent revisions of the manuscript, data analysis, and final draft of the manuscript. N.K contributed in the literature search and A.K.R. assisted in figure preparation and data collection. P.G. performed the literature search.

## Conflicts of interest

None declared.

## Financial disclosure

No specific grant was obtained from any funding agency in public, commercial, or not-for-profit sectors.

## References

[1] Pappas PG (2013). Cryptococcal infections in non-HIV-infected patients. Trans Am Clin Climatol Assoc.

[2] Kwon-Chung KJ, Varma A (2006). Do major species concepts support one, two or more species within Cryptococcus neoformans?. FEMS Yeast Res.

[3] Maziarz EK, Perfect JR (2016). Cryptococcosis. Infect Dis Clin N Am.

[4] Spies FS, de Oliveira MB, Krug MS, Severo CB, Severo LC, Vainstein MH (2015). Cryptococcosis in patients living with hepatitis C and B viruses. Mycopathologia.

[5] Suchitha S, Sheeladevi CS, Sunila R, Manjunath GV (2012). Disseminated cryptococcosis in an immunocompetent patient: a case report. Case Rep Pathol.

[6] Subramanian S, Mathai D (2005). Clinical manifestations and management of cryptococcal infection. J Postgrad Med.

[7] El-Serag HB, Anand B, Richardson P, Rabeneck L (2003). Association between hepatitis C infection and other infectious diseases: a case for targeted screening?. Am J Gastroenterol.

[8] Gupta KL (2001). Fungal infections and the kidney. Indian J Nephrol.

[9] National viral hepatitis control program (NVHCP) (2018). Ministry of Health and Family Welfare (MoHFW), Government of India.

[10] Aye C, Henderson A, Yu H, Norton R (2016). Cryptococcosis-the impact of delay to diagnosis. Clin Microbiol Infect.

[11] Bratton EW, El Husseini N, Chastain CA, Lee MS, Poole C, Stürmer T (2012). Comparison and temporal trends of three groups with cryptococcosis: HIV-infected, solid organ transplant, and HIV-negative/non-transplant. PLoS One.

